# ITIH5 as a multifaceted player in pancreatic cancer suppression, impairing tyrosine kinase signaling, cell adhesion and migration

**DOI:** 10.1002/1878-0261.13609

**Published:** 2024-02-20

**Authors:** Jennifer Kosinski, Antonio Sechi, Johanna Hain, Sophia Villwock, Stefanie Anh Ha, Maximilian Hauschulz, Michael Rose, Florian Steib, Nadina Ortiz‐Brüchle, Lara Heij, Sanne L. Maas, Emiel P. C. van der Vorst, Thomas Knoesel, Annelore Altendorf‐Hofmann, Ronald Simon, Guido Sauter, Jan Bednarsch, Danny Jonigk, Edgar Dahl

**Affiliations:** ^1^ Institute of Pathology Medical Faculty of RWTH Aachen University Germany; ^2^ Center for Integrated Oncology Aachen Bonn Cologne Duesseldorf (CIO ABCD) Germany; ^3^ Department of Cell and Tumor Biology RWTH Aachen University Germany; ^4^ Institute of Pathology University Hospital Essen Germany; ^5^ Department of Surgery and Transplantation, Medical Faculty RWTH Aachen University Germany; ^6^ Department of Pathology Erasmus Medical Center Rotterdam The Netherlands; ^7^ NUTRIM School of Nutrition and Translational Research in Metabolism Maastricht University The Netherlands; ^8^ Interdisciplinary Center for Clinical Research (IZKF), Institute for Molecular Cardiovascular Research (IMCAR) Medical Faculty of RWTH Aachen University Germany; ^9^ Institute for Cardiovascular Prevention (IPEK) Ludwig‐Maximilians‐University Munich Germany; ^10^ Institute of Pathology Ludwig‐Maximilians‐University Munich Germany; ^11^ Department of General, Visceral and Vascular Surgery University Hospital Jena Germany; ^12^ Institute of Pathology University Medical Center Hamburg‐Eppendorf Germany; ^13^ RWTH centralized Biomaterial Bank (RWTH cBMB) Medical Faculty of the RWTH Aachen University Germany; ^14^ German Center for Lung Research (DZL), BREATH Hanover Germany

**Keywords:** C2TSG, focal adhesions, ITIH5, metastasis suppressor, pancreatic cancer, tumor cell migration

## Abstract

Inter‐alpha‐trypsin inhibitor heavy chain 5 (*ITIH5*) has been identified as a metastasis suppressor gene in pancreatic cancer. Here, we analyzed *ITIH5* promoter methylation and protein expression in The Cancer Genome Atlas (TCGA) dataset and three tissue microarray cohorts (*n* = 618), respectively. Cellular effects, including cell migration, focal adhesion formation and protein tyrosine kinase activity, induced by forced *ITIH5* expression in pancreatic cancer cell lines were studied in stable transfectants. *ITIH5* promoter hypermethylation was associated with unfavorable prognosis, while immunohistochemistry demonstrated loss of ITIH5 in the metastatic setting and worsened overall survival. Gain‐of‐function models showed a significant reduction in migration capacity, but no alteration in proliferation. Focal adhesions in cells re‐expressing *ITIH5* exhibited a smaller and more rounded phenotype, typical for slow‐moving cells. An impressive increase of acetylated alpha‐tubulin was observed in ITIH5‐positive cells, indicating more stable microtubules. In addition, we found significantly decreased activities of kinases related to focal adhesion. Our results indicate that loss of ITIH5 in pancreatic cancer profoundly affects its molecular profile: ITIH5 potentially interferes with a variety of oncogenic signaling pathways, including the PI3K/AKT pathway. This may lead to altered cell migration and focal adhesion formation. These cellular alterations may contribute to the metastasis‐inhibiting properties of ITIH5 in pancreatic cancer.

## Graphical abstract

*ITIH5* expression in the absence of promoter hypermethylation influences intracellular processes. These include inhibition of various tyrosine kinases, remodeling of the cytoskeleton and impairment of focal adhesion formation. This complex interplay, observed *in vitro* after forced *ITIH5* re‐expression, may clinically been reflected by a survival benefit of pancreatic cancer patients with sustained *ITIH5* expression.

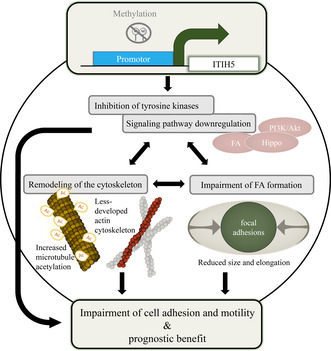

**Abbreviations**
ACTB: actin beta gene
BSA: bovine serum albumin
C2TSG: Class 2 tumor suppressor gene
CIMP: CpG island methylator phenotype
CIO ABCD: Center for Integrated Oncology Aachen Bonn Cologne Duesseldorf
CLB: cytoskeleton lysis buffer
CT: cycle threshold
DAPI: 4′,6‐diamidino‐2‐phenylindole
DNA: desoxyribonucleic acid
DTT: dithiothreitol
ECM: extracellular matrix
EDTA: ethylenediaminetetraacetic acid
EGTA: ethylene glycol tetraacetic acid
EK: ethical committee
FA: focal adhesion
FBS: fetal bovine serum
FDR: false discovery rate
FFPE: formalin‐fixed paraffin‐embedded
FITC: fluorescein
FOLFIRNOX: fluorouracil/leucovorin, irinotecan und oxaliplatin
GO: gene ontology
HA: hyaluronic acid
HE: hematoxylin and eosin
ID: identification
IMCAR: Institute for Molecular Cardiovascular Research
IPEK: Institute for Cardiovascular Prevention
ITI: inter‐alpha‐trypsin inhibitor
ITIH5: inter‐alpha‐trypsin inhibitor heavy chain 5
IZKF: Interdisciplinary Center for Clinical Research
LMU: Ludwig‐Maximilians‐Universität München
MOPS: 3‐(*n*‐morpholino)propanesulfonic acid
mRNA: messenger ribonucleic acid
ORA: over‐representation analyses
PBS: phosphate‐buffered saline
PDAC: pancreatic adenocarcinoma
PFS: progression‐free survival
PI3K: phosphoinositide‐3‐kinase
PTK: peptide tyrosine kinase
PY20: phosphotyrosine monoclonal antibody
RIPA: radio immunoprecipitation assay
RNA: ribonucleic acid
RTK: receptor tyrosine kinase
RT‐PCR: real‐time polymerase chain reaction
RWTH: Rheinisch‐Westfälische Technische Hochschule Aachen
RWTH cBMB: RWTH centralized biomaterial bank
SDS: sodium dodecyl sulfate
SEM: standard of the mean
SNP: single nucleotide polymorphism
SPSS: Statistical Package for the Social Sciences
TCGA: The Cancer Genome Atlas
TIRF: total internal reflection fluorescence
TMA: tissue microarray
UICC: Union for International Cancer Control
XTT: 2,3‐bis‐(2‐methoxy‐4‐nitro‐5‐sulfophenyl)‐2 *h*‐tetrazolium‐5‐carboxanilide

## 1 Introduction

Inter‐alpha‐trypsin inhibitor heavy chain 5 (ITIH5) is a member of the Inter‐α‐trypsin inhibitor (ITI) protein family that belongs to the group of secreted serine protease inhibitors and occurs in the blood circulation and the extracellular matrix (ECM) [1]. ITI molecules are composed of one light chain (bikunin) and one to two covalently bound homolog heavy chains [1, 2]. Bikunin holds the serine protease inhibitory activity [1], whereas ITIHs, including ITIH1‐ITIH5 [1, 3], can stabilize the ECM [4] by covalently binding hyaluronic acid (HA) and connecting multiple HA molecules to cable‐like structures [5, 6]. Therefore, it is plausible to assume that loss of ITIHs, as it often occurs during carcinogenesis of solid tumors, has a negative effect on ECM stabilization and thus promotes tumor growth [7] and metastasis (see below). Indeed, numerous studies have shown that ITIH5 loss influences cancer progression and thus patient prognosis [8, 9, 10, 11, 12, 13, 14]. With respect to metastasis suppression of ITIHs in various entities, ITIH1 and ITIH3 were shown to reduce metastasis in lung cancer [15], whilst metastasis suppressive effects of ITIH5 were described for breast [16, 17], bladder [11] and pancreatic cancer [18]. Interestingly, in breast cancer, ITIH5 has been shown to suppress both tumor proliferation and metastasis, while in pancreatic cancer, ITIH5 is considered primarily a metastasis suppressor. This assessment is based on the observation that forced expression of ITIH5 in pancreatic cancer cell lines and in a mouse model of liver metastases led to reduced cell motility and invasion but did not reduce proliferation [18, 19]. Within the ITIH gene family, ITIH5 takes on a distinctive position as it is the only ITIH gene being silenced by promoter DNA hypermethylation. ITIH5 promoter hypermethylation was described in different tumor entities [8, 10, 11, 20, 21] and at least in breast [8], bladder [11], colon [13], and lung cancer [21], this event was linked to unfavorable prognosis. Because of this connection between ITIH5's epigenetic silencing in cancer and a resulting unfavorable prognosis, ITIH5 is now being regarded as a class 2 tumor suppressor gene (C2TSG) [22] or DNA methylation driver [23]. The characterization of ITIH5 as a C2TSG in pancreatic cancer has not yet been investigated, but its significance could emerge in the future. C2TSGs hold promise as potential targets for novel therapeutic approaches [24], especially given that promoter methylation in the context of general epigenetic changes plays a pivotal role in altered gene expression and defining the malignant phenotypes of pancreatic cancer [25].

Pancreatic ductal adenocarcinoma (PDAC) is the leading pancreatic malignancy, and it is one of the deadliest malignancies accounting for almost as many deaths as cases worldwide [26, 27]. It is the third leading cause of cancer‐related deaths in the United States [28] and the second leading cause in Germany [29]. Despite a modest improvement in the five‐year survival rate over the recent decades, it remains remarkably low at 11% [28]. This poor prognosis is due to several clinical and molecular biological challenges in PDAC that make its treatment difficult, including nonspecific symptoms, early metastasis, and limited diagnostic capabilities as well as lacking therapeutic strategies [30]. Therapeutic options are severely restricted due to resistance to chemotherapy [30] and the heterogeneous nature of PDAC [25]. Consequently, microscopically margin‐negative resection (R0) at the primary tumor site remains the sole curative therapy option [26, 31]. Therefore, better strategies and a better molecular understanding of PDAC heterogeneity are needed to face this challenging tumor entity and advance more targeted approaches.

In this study, we wanted to decipher whether and how ITIH5 may influence the hallmarks of cancer in PDAC and which signaling pathways and cellular structures may be affected after forced re‐expression of ITIH5 in PDAC cell lines. Given the metastasis‐suppressing properties of ITIH5 in pancreatic cancer, we focused on further investigating the impact on focal adhesions, as these cellular structures have a profound influence on cell migration [32]. In particular, the influence of ITIH5 expression on focal adhesions and consequently reduced migratory capacity has been described previously in bladder cancer [33]. To gain further insight into the underlying molecular mechanisms responsible for the effects of ITIH5 overexpression, we conducted a profiling of 196 protein tyrosine kinases (PTKs). This profiling enabled us to assess the activity of multiple PTKs that play crucial roles in cellular signaling pathways, aiming to potentially identify specific pathways that are affected by forced ITIH5 re‐expression in PDAC cells.

## 2 Materials and methods

### 2.1 Genome‐wide methylation analysis using TCGA data sets data retrieval

Infinium HumanMethylation450 BeadChip and RNASeqV2 data of pancreatic cancer (PANC) and corresponding normal tissue samples were obtained from The Cancer Genome Atlas (TCGA) data portal (*n* = 177, for sample IDs see Table S1) and can be explored using the cBio Cancer Genomics Portal (http://cbioportal.org) [34, 35]. Beta values of CpG sites close to the *ITIH5* promoter (CG09445472, CG01382938, CG1011975, CG10151473, CG04711998, CG22444507, CG10311806) were used to visualize tumor‐specific DNA hypermethylation.

### 2.2 Study populations, tumor samples, and ethical approval

The study population from Aachen was obtained from 41 patients with pancreatic ductal adenocarcinoma (PDAC), who received surgical treatment at the Department of General and Visceral Surgery at the University Hospital RWTH Aachen (Aachen, Germany) between 2012 and 2014. The clinico‐pathological characteristics are shown in Table S2. Only pancreatic resections, not biopsies, were considered. For each case, clinical data (age, sex, UICC classification 7th edition 2010) were summarized from histopathological reports. Formalin‐fixed paraffin‐embedded (FFPE) primary tumor and metastases tissue samples of the PDAC cases were acquired from the archive of the Institute of Pathology of RWTH Aachen University Hospital. A tissue microarray (TMA) consisting of PDAC tumor and metastases tissues with a diameter of 2 mm, was constructed using a semiautomatic TMArrayer™ (Pathology Devices).

The Institute for Pathology of the University Hospital Hamburg provided a TMA of different pancreatic malignant tissues (years 1993–2005), including 357 primary tumors, 129 corresponding lymph node metastases and 22 distant metastases. For this study, only ductal adenocarcinomas (*n* = 199) with their corresponding metastases (*n* = 65) were used. The exact description of the TMA and the clinical characteristics of the cohorts have been described previously [36], the clinico‐pathological characteristics are summarized in Table S3.

The Institute of Pathology of the University Hospital Jena and the Pathology Institute of the LMU Munich provided TMA sections containing 378 tissue samples of pancreatic ductal adenocarcinomas (years 1995–2012). The exact description of the TMA and the clinical characteristics of the cohorts have been described previously [37]. Clinical follow‐up data like survival time, sex, age, and adjuvant chemotherapies were collected by the prospective tumor registry of the Department of Surgery of the University Hospital Jena and the Munich Cancer Registry.

This retrospective study was conducted in accordance with the Declaration of Helsinki. All study cohorts received ethical approvals from the respective ethics committees, i.e. at the Medical Faculty of RWTH Aachen University (ethical vote EK 100/21), at the Ärztekammer Hamburg (ethical vote WF‐049/09) and at the LMU Munich University hospital (ethical vote 307‐16 UE). Due to the work with diagnostic material approved by the local ethics committees, the individual consent of the patient was waived.

### 2.3 Immunohistochemistry

Paraffin sections (2 μm) of formalin‐fixed tissues were dewaxed and rehydrated with a pT‐Link pretreatment module (Agilent, Santa Clara, CA, USA). Immunohistochemical staining of the sections was performed following the manufacturer's instructions with a Lab Vision™ Autostainer 360‐2D (Thermo Fisher Scientific, Waltham, MA, USA) using an anti‐ITIH5 rabbit polyclonal primary antibody (generated by Pineda Berlin antibody service; dilution 1 : 200). The antigen and antibody complexes were visualized with the EnVision™ Flex System (Agilent) using horseradish peroxidase and the substrate diaminobenzidine. The sections were counterstained with haemalaun, dehydrated by an ascending alcohol series and manually covered. One section without primary antibody incubation always served as the technical negative control. Placenta and kidney tissue, known to have a high and medium ITIH5 expression respectively, served as positive controls. The expression level of ITIH5 was scored by experienced pathologist according to a four‐tier scale (0 = negative; 1 = weak; 2 = moderate; 3 = strong).

### 2.4 FFPE patient cohort

For FFPE patient cohort, a previously described study population from Aachen was analyzed [38], including FFPE tissue samples of 28 PDAC patients with corresponding adherent normal acinar pancreas tissues and seven corresponding metastases. Briefly, as prior described in [38], 2 μm sections were prepared for each tissue sample and stained with hematoxylin and eosin (HE) and assessed by an experienced pathologist to highlight relevant areas of the tissues.

### 2.5 Nucleic acid extraction from FFPE tissues and cell lines

Sections of FFPE tissue were processed as previously described [38]. Genomic DNA from FFPE tissue and from cell lines was isolated using the QIAamp^®^ DNA Mini kit (Qiagen, Hilden, Germany) according to the manufacturer's instructions. Total cellular RNA from cultured cells was extracted using NucleoSpin RNA plus (Macherey‐Nagel, Düren, Germany) according to the manufacturer's instructions likewise. RNA from FPPE tissue was extracted using the ReliaPrep™ FFPE Total RNA Miniprep System (Promega) as previously described [38]. The concentration of nucleic acids was measured with the NanoDrop ND‐1000 Spectrophometer (VWR). For long‐term storage, samples were stored at −80 °C.

### 2.6 Reverse transcription and real‐time polymerase chain reaction (RT‐PCR)

Complementary DNA (cDNA) of 1 μg of each total RNA sample was synthesized using the Reverse Transcription System (Promega). Amplification of cDNA by RT‐PCR using iTaq™ Universal SYBR Green Supermix (Bio‐Rad Laboratories) was performed as previously described [39] using the CFX96 Touch Real‐Time PCR System (Bio‐Rad Laboratories). Relative gene expression was quantified using the comparative CT method [40]. The housekeeping gene β‐actin (ACTB) was used as a reference. All used primers span at least one intron and are listed in Table S4.

### 2.7 DNA bisulfite conversion and bisulfite pyrosequencing

For DNA bisulfite conversion 500 ng of genomic DNA was processed for 16 h using the EZ DNA Methylation™ kit (Zymo Research, Freiburg, Germany) according to the manufacturer's instructions. Subsequent pyrosequencing was performed by using the PyroMark Q96 ID sequencer (Qiagen) with PyroMark PCR Kit, PyroMark Gold Q96 Reagents, PyroMark Annealing Buffer and PyroMark Wash Buffer (Qiagen) as components. For analysis, a previously designed ITIH5 pyrosequencing assay was used [41]. Used primers are listed in the Table S5. Primers and sequence of interest meet similar criteria based on TCGA data analysis as previously described [41]: The sequences of interest should compromise promoter regions that are (a) characterized by strong differences in mean DNA methylation between pancreatic normal and pancreatic cancer samples and (b) are located in important gene regulatory sequences. As positive controls for unmethylated and methylated DNA, the EpiTect^®^PCR Control DNA Set (Qiagen) was used. Any methylation significantly higher than the average methylation in normal tissue was regarded as hypermethylation.

### 2.8 Cell lines and cell culture

The human PDAC cell lines PANC‐1 (RRID:CVCL_0480), MIA PaCa‐2 (RRID:CVCL_0428), BxPC‐3 (RRID:CVCL_0186) and AsPC‐1 (RRID:CVCL_0152) were provided by the Department of General, Visceral and Transplant Surgery, University Hospital RWTH Aachen. PSN1 (RRID:CVCL_1644) and DAN‐G (RRID:CVCL_0243) cell lines were provided by the Institute of Pathology, University Hospital HHU Düsseldorf. Cells were cultured under ideal conditions (37 °C, 5 vol‐% CO_2_, 20% O_2_, 95% humidity). PANC‐1 and MIA PaCa‐2 cells were cultivated in GibcoTM DMEM high glucose medium and PSN‐1, BxPc‐3, AsPC‐1 and DAN‐G cells in GibcoTM RPMI 1640 medium. Each medium was supplemented with 10% fetal bovine serum, 1 mm pyruvate, 50 U mL‐1 penicillin, 50 mg mL^−1^ streptomycin and 2 mm L‐glutamine. Single nucleotide polymorphism (SNP) profiling for authentication was performed on all cell lines provided by Multiplexion and all cell lines were regularly tested for mycoplasma infection.

### 2.9 Western blot

For western blot analysis, cultured cells were washed in PBS and extracted in NuPage LDS sample buffer (Invitrogen, Carlsbad, CA, USA) under reducing conditions (50 mm DTT). Subsequent sonification using Sonopuls (Bandelin, Berlin, Germany) enabled disruption and release of intracellular proteins. Equal protein concentrations were processed as previously described [42]. Accordingly, the cytoplasmatic proteins were separated in 4–12% Bis‐Tris gels (Invitrogen) using MOPS‐SDS running buffer and electroblotted onto nitrocellulose membranes (0.2 μm). Used primary antibodies are listed in Table S6. The anti‐ITIH5 antibody has already previously been characterized. [11] Protein signal of the housekeeping gene β‐actin was used for normalization.

### 2.10 Transfection and single‐cell cloning of PANC‐1 and PSN‐1 cells

PANC‐1 and PSN‐1 cells were transfected with either the ITIH5‐pBK‐CMV expression vector containing the empty vector (mock clones) or the full‐length human *ITIH5* cDNA (*ITIH5* clones) as previously described [3]. Transfection was performed by using the FuGENE® HD transfection reagent (Promega) following the manufacturer's instructions. For transfection, a ratio of transfection reagent to DNA of 3 : 1 for PANC‐1 and 4 : 1 for the PSN‐1 cell line was used. Single‐cell clones were selected by the antibiotic geneticin [Geneticin (G418); Invitrogen; PANC‐1: 1000 μg·mL^−1^; PSN‐1: 400 μg·mL^−1^]. For cell culture assays, two mock and two ITIH5 clones were selected for each cell line and three independent runs were performed.

### 2.11 Cell count assay

Stable transfected PANC‐1 ITIH5 and mock clones were seeded as triplicates in 6‐well plates (2 × 10^4^ cells per well). PSN‐1 stable ITIH5 and mock clones were seeded as triplicates in 12‐well plates (1 × 10^4^ cells per well). Cell number of viable cells was determined 24, 48, 72, and 96 h after cell seeding by using the CASY‐1 cell counter and analyzer system (Schärfe System, Reutlingen, Germany).

### 2.12 XTT cell proliferation assay

Stable PANC‐1 and PSN‐1 ITIH5 and mock clones were investigated with the XTT Cell Proliferation Kit II (Roche Diagnostics, Rotkreuz, Switzerland). The assay was performed as previously described [8].

### 2.13 Apoptosis assay

The activities of the effector caspases 3 and 7 in stable PANC‐1 and PSN‐1 ITIH5 and mock clones were analyzed by using the Apo‐One^®^ Homogeneous Caspase‐3/7 Assay (Promega) as previously described [16], but slightly modified by seeding 2 × 10^4^ cells and using 0.5 μm staurosporine (Sigma‐Aldrich) to induce apoptosis.

### 2.14 Colony formation assay

PANC‐1 and PSN‐1 ITIH5 and mock clones were seeded as triplicates in 6‐well plates (1 × 10^3^ cells per well). Cells were incubated under ideal culture conditions and geneticin selection pressure for 10 days. After cultivation, colonies were fixed with 3.5% formaldehyde/80% methanol and stained with 0.1% crystal violet for 30 min. Colonies were photographed (Gel Jet Imager, Intas) and densitometric analysis was performed using fiji [43].

### 2.15 Wound healing assay

PANC‐1 and PSN‐1 ITIH5 and mock clones were seeded in silicon inserts (Ibidi) and allowed to adhere overnight (PANC‐1: 8 × 10^4^ cells per insert; PSN‐1: 6 × 10^4^ cells per insert). Cells were incubated with Mitomycin C to inhibit cell proliferation (PANC‐1: 10 μg·mL^−1^, 30 min, PSN‐1: 20 μg·mL^−1^, 1 h). After insert removal, the closure of the generated gap was microscopely captured at 0, 24, and 48 h for the PANC‐1 and at 0, 16, 24, and 40 h for the PSN‐1 cell line using the Axiovert 100 TV Microscope (Zeiss, Jena, Germany). For wound closure quantification, images were processed and analyzed with fiji using a plugin generated for image analysis of *in vitro* scratch wound healing assays [44]. For live cell imaging, cell migration during a wound healing assay was recorded for 24 h using an Axio observer Z1 inverted microscope equipped with a heating stage, CO2 controller and an Evolve EM‐CCD camera driven by zen software (Zeiss). Images were acquired every 5 min and final videos were assembled using fiji.

### 2.16 Transwell migration and invasion assay

The assays were conducted in 24‐well plates carrying transwell inserts with microporous 8 μm membranes for migration assay and Matrigel‐coated microporous 8 μm membranes for invasion assay Corning (Corning, NY, USA). PANC‐1 and PSN‐1 ITIH5 and mock clones suspended in FBS‐free medium were seeded in the upper chamber (5 × 10^5^ cells per insert). Medium containing 20% FBS in the lower chamber served as the chemoattractant. After 24 h of incubation under ideal culture conditions, transwell inserts were transferred to a new 24‐well plate. Non‐migrated or non‐invaded cells were removed from the top of the membrane. The adhered cells on the bottom of the membrane were fixed with 3.5% formaldehyde/80% methanol and stained with 0.1% crystal violet for 30 min. Membranes were washed with water and mounted on microscope slides. Migrated or invaded cells were counted in four representative fields with the Axioplan 2 Microscope (Zeiss, 10× magnification).

### 2.17 Immunofluorescence microscopy

Immunofluorescence labeling was done as previously described [45, 46, 47]. Briefly, PANC‐1 and PSN‐1 cells were fixed with 4% PFA in cytoskeleton buffer pH 7.0 for 20 min. at room temperature. After washing once with PBS, cells were permeabilized with 0.1% Triton X‐100 for 1 min. at room temperature followed by three washing steps with PBS. For talin labeling, cells were fixed with ice‐cold (−20 °C) methanol for 4 min. on ice, rehydrated with PBS containing 0.1% Triton X‐100 (3 times, 5 min. each), and finally washed with PBS.

Focal adhesions were visualized using antibodies against vinculin (hVIN1, Sigma‐Aldrich, St. Louis, MO, USA, diluted 1 : 200), zyxin (ABC1387, Sigma‐Aldrich, diluted 1 : 500), paxillin (clone 5H11, Thermo Fisher Scientific, diluted 1 : 200) or talin (14168‐1‐AP, Proteintech, diluted 1 : 200) followed by Alexa 594‐conjugated anti‐mouse or anti‐rabbit IgG (Thermo Fisher Scientific, diluted 1 : 500). The actin cytoskeleton was visualized using Alexa 488‐conjugated phalloidin (Thermo Fisher Scientific, diluted 1 : 300). Nuclei were stained using DAPI (diluted 1 : 1000).

Images were acquired using an Axio Observer Z1 equipped with an Evolve electron‐multiplying charge‐coupled device camera (Teledyne Photometrics, Tucson, AZ, USA) driven by ZEN software (Zeiss). Total internal reflection fluorescence (TIRF) microscopy was done using an Axio Observer Z1 equipped with a motorized TIRF slider. Excitation of Alexa 488 and Alexa 594 was done using 488 and 561 nm laser lines (both at 10% of their power), respectively. The depth of the evanescent field for both wavelengths was ~ 70 nm. Digital handling of the images was done using ZEN (Zeiss), fiji, and Adobe Photoshop (Adobe Systems, Mountain View, CA, USA).

### 2.18 Analysis of focal adhesion static properties

The analysis of focal adhesion was done using a dedicated segmentation algorithm as already described [48, 49, 50]. The following static focal adhesion parameters were determined: area, major axis, minor axis, and elongation (ratio major/minor axis). For each PANC‐1 and PSN‐1 clone, at least 100–150 focal adhesions were analyzed (PANC‐1: *n*_ITIH5 13_ = 135, *n*_ITIH5 23_ = 127, *n*_mock 1_ = 131, *n*_mock 4_ = 145; PSN‐1: *n*_ITIH5 11_ = 146, *n*_ITIH5 12_ = 157, *n*_mock 1_ = 121, *n*_mock 3_ = 175).

### 2.19 Quantification of F‐actin and acetylated alpha‐tubulin

Following the immunofluorescence labeling as described in the section above, for the quantification of F‐actin and acetylated alpha‐tubulin, up to 10 confocal images/stacks of different fields of interest were acquired using an LSM 700 confocal microscope equipped with a 63× oil immersion objective and 405 and 555 nm laser lines (Zeiss). The 405 nm laser line was used to excite DAPI, whereas the 555 nm laser line was used to excite Alexa 594 fluorophores. Confocal settings such as laser intensity, gain and pinhole aperture were kept constant for all images. Image processing was done using fiji. After selecting regions of interest as closely as possible corresponding to the shape of single cells, the average pixel intensity was calculated. To normalize the quantification, the average pixel intensity of background (not containing cells) regions of interest was subtracted from each measurement. Plots and statistics were done using prism 10 (GraphPad Software, La Jolla, CA, USA).

### 2.20 Kinase activity profiling

Tyrosine kinase profiles were determined using the PamChip^®^ peptide kinase microarray system with the PamStation^®^12 (PTK; PamGene International) as previously described [51, 52]. In brief, cells were cultured as described in 2.8 and washed once in ice‐cold PBS after respective treatments, with four biological replicates per condition, and lysed for 15 min. on ice using M‐PER Mammalian Extraction Buffer containing Halt Phosphatase Inhibitor and EDTA‐free Halt Protease Inhibitor Cocktail (1 : 100 each; Thermo Fischer Scientific). Lysates were centrifuged for 15 min. at 16 000 ***g*** at 4 °C in a pre‐cooled centrifuge. Protein quantification was performed with Pierce™ Bradford Plus Protein Assay (Thermo Fisher Scientific) Assay according to the manufacturer's instructions.

For the PTK assay, 10.0 μg of protein was applied per array (*n* = 4 per condition) and carried out using the standard protocol supplied by Pamgene. PTK Basic Mix was prepared as described [51], by adding the freshly frozen lysate to 4 μL of 10× protein PTK reaction buffer (PK), 4 μL of 10× PTK additive, 4 μL of 4 mm ATP, 0.4 μL of 100× bovine serum albumin (BSA), 0.4 μL of 1 m dithiothreitol (DTT) solution and 0.6 μL of monoclonal anti‐phosphotyrosine FITC‐conjugate detection antibody (clone PY20). Total volume of the PTK Basic Mix was adjusted to 40 μL by adding distilled water (H_2_0). Prior to loading the PTK Basic Mix onto the array, a blocking step was performed in which 30 μL of 2% BSA was applied to the center of each array and washed with PTK solution for PamChip^®^ pre‐processing. Next, 40 μL of PTK Basic Mix was applied to each array of the PamChips^®^. The microarray assays were run for 94 cycles. Finally, an image was captured by a CCD camera PamStation^®^12 at kinetic read cycles 32–93 at 10, 50, and 200 ms and at end‐level read cycle at 10, 20, 50, 100, and 200 ms. Spot intensity at each time point was quantified (and corrected for local background) using bionavigator software version 6.3 (PamGene International). Upstream Kinase Analysis [53], a functional scoring method (PamGene) was used to rank kinases based on combined specificity scores (based on peptides linked to a kinase, derived from 6 databases) and sensitivity scores (based on treatment‐control differences). The list of kinases of interest contains the kinases with higher median final scores (> 1.2). The *P*‐values were adjusted for multiple comparisons by false discovery rate (FDR). Over‐representation analyses (ORA) of WikiPathways for the kinases with significant differences (median final scores > 1.2 and adjusted *P*‐value < 0.05) from baseline were performed using the clusterprofiler r‐package [54].

### 2.21 Statistics

Statistical analyses were performed using spss 27.0 (IBM, Armonk, NY, USA) for patient cohort analyses and graphpad prism 10.0 (GraphPad Software) for the cell culture experiments. Statistical significance was defined as a *P*‐value < 0.05. Normality distribution of the data sets was tested using the Shapiro–Wilk test. To compare two groups the non‐parametric Mann–Whitney *U* test was used. To compare more than two groups One‐Way Analysis of Variance (ANOVA) was used. In case of multiple group comparison, Turkey's multiple comparison test was performed as *post hoc* test. Immunohistochemistry data comparing tumor and metastasis was considered as unmatched as data for metastasis was incomplete. Overall survival time was calculated from the date of pancreatic surgery to death, irrespective of its cause. Survival curves were calculated by the Kaplan–Meier method and the log‐rank test was used to assess differences in survival. All error bars indicate the standard error of the mean (SEM).

## 3 Results

### 3.1 *ITIH5* promoter hypermethylation is associated with unfavorable overall survival in PDAC of the TCGA cohort

**Fig. 1:**
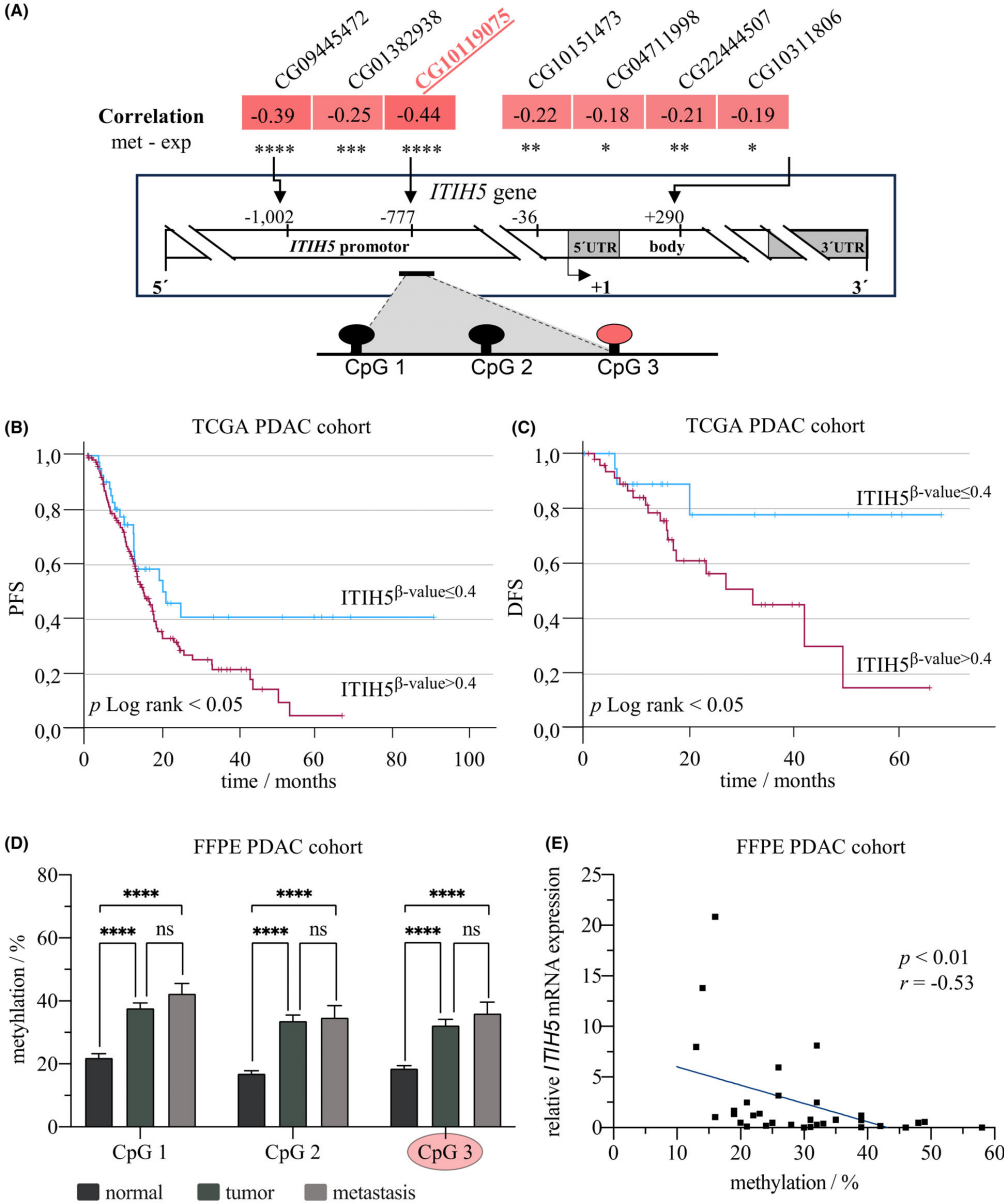
*ITIH5* promoter methylation analysis in The Cancer Genome Atlas (TCGA) dataset and hypermethylation in independent formalin‐fixed paraffin‐embedded (FFPE) tissue cohort. (A) Schematic representation of the *ITIH5* gene with focus on its promoter region (UTR = untranslated region). TCGA data of seven selected CpG sites demonstrate a significant anti‐correlation between *ITIH5* promoter methylation (met) and *ITIH5* mRNA expression (exp) (Spearman's rank correlation coefficient). Lollipop cartoon illustrating the localization of the three CpG sites covered by the pyrosequencing assay marked by a black line including CG10119075 (red dot, CpG3). (B) Kaplan–Meier plot (log‐rank test). Progression‐free survival (PFS) of all pancreatic adenocarcinoma (PDAC) patients combined (*n* = 176, events = 102, *P* < 0.05) is significantly longer in the case of low *ITIH5* methylation (β‐value of CG10119075 ≤ 0.4) and thus higher ITIH5 expression. (C) Kaplan–Meier plot (log‐rank test). Disease‐free survival (DFS) of all PDAC patients combined (*n* = 68, events = 22, *P* < 0.05) is significantly longer in the case of low *ITIH5* methylation (β‐value of CG10119075 ≤ 0.4) and thus higher ITIH5 expression. (D) Hypermethylation of analyzed CpG sites in tumors (*n* = 28) and metastases (*n* = 7) compared to normal tissue (*n* = 28) in a FFPE tissue cohort [One‐Way Analysis of Variance (ANOVA), Turkey's multiple comparison test]. (E) Significant anti‐correlation of overall *ITIH5* promoter methylation and relative *ITIH5* mRNA expression (Spearman's rank correlation coefficient, *r* = −0.53, *P* < 0.01) in FFPE tissue cohort. Error bars are indicating the standard error of the mean (SEM), ns = not significant, * < 0.05, ** < 0.01, *** < 0.001, **** < 0.0001.

To determine promoter methylation in the pancreatic cancer cases of the TCGA dataset, seven CpG sites in the *ITIH5* promoter were analyzed in analogy to a previous analysis of our research group in bladder cancer [41]. All seven CpG sites showed significant hypermethylation in pancreatic cancer compared to normal controls. The highest inverse correlation between ITIH5 expression and *ITIH5* promoter methylation was found at CpG site CG10119075 (Fig. 1A, *R* = 0.44, *P* < 0.001), which is located at position −777 relative to the *ITIH5* gene transcription start point. This region (−777 to −1002 including CpG1, CpG2 and CpG3) was found to be particularly relevant for *ITIH5* transcriptional regulation in bladder cancer as well [41]. Measured across all pancreatic cancer patients in the TCGA cohort, a methylated CG10119075 CpG site was associated with both significantly shorter progression‐free survival (Fig. 1B, *P* < 0.05) as well as disease‐free survival (Fig. 1C, *P* < 0.05). Thus, these data clearly indicate that high *ITIH5* promoter methylation in pancreatic cancer is associated with (yet increased) unfavorable prognosis.

### 3.2 *ITIH5* promoter is robustly hypermethylated in pancreatic tumors and metastasis

Subsequently to the analysis of the TCGA cohort, we used a recently described PDAC patient cohort from our hospital [38] and analyzed it for *ITIH5* promoter hypermethylation. The cohort included matched patient tissues with 28 normal, 28 tumor, and 7 metastasis samples. DNA was isolated and pyro‐sequenced, adopting the assay design from our previous work on bladder cancer [41], which included the CG10119075 CpG site mentioned in 3.1 (Fig. 1A). All three CpG sites analyzed showed significantly increased promoter methylation in tumor and metastatic tissue compared to normal tissue (Fig. 1D, *P* < 0.0001), including significantly greater DNA methylation of CpG site CG10119075 in metastases (M = 0.38, SEM = 0.04, *P* < 0.0001) and tumor tissues (M = 0.36, SEM = 0.02, *P* < 0.0001) compared to normal tissues (M = 0.19, SEM = 0.01) (Fig. 1D). In contrast, comparing metastasis and tumor samples, no significant differences in promoter methylation were seen (Fig. 1D). In addition to DNA extraction, RNA was successfully isolated from a subset of this PDAC patient cohort, which included 13 normal, 14 tumors and 5 metastases. The corresponding data showed an inverse correlation between *ITIH5* promoter methylation and ITIH5 mRNA expression levels (Fig. 1E, *R* = −0.53, *P* < 0.01). Hence, analysis of our own patient cohort confirmed the findings from TCGA data, i.e., significant *ITIH5* promoter hypermethylation in pancreatic cancer is accompanied by simultaneous loss of ITIH5 mRNA expression.

### 3.3 Downregulation of ITIH5 expression in pancreatic cancer in the metastatic setting

**Fig. 2:**
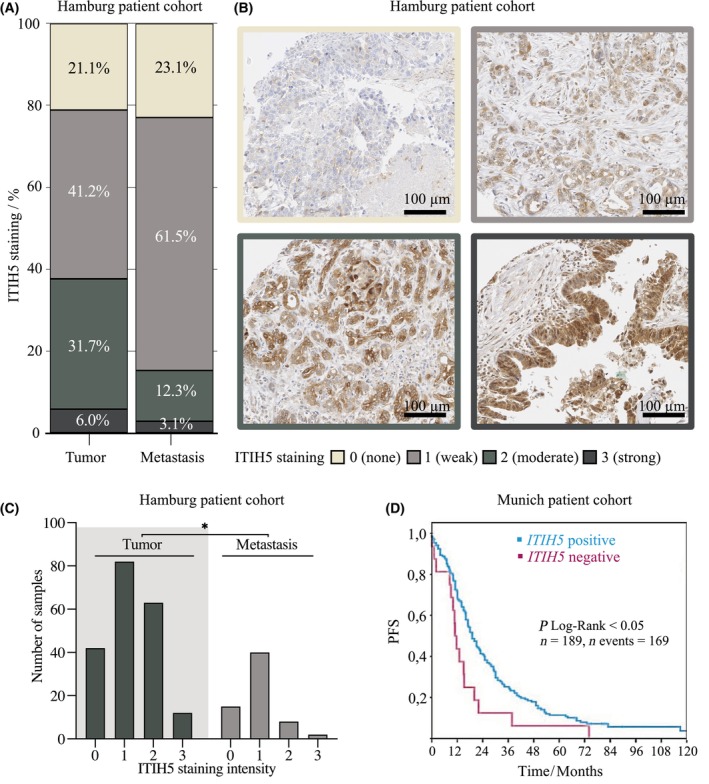
Loss of ITIH5 expression in pancreatic adenocarcinoma (PDAC) metastases and prognostic advantage of ITIH5 expression. (A) Distribution of ITIH5 expression in the Hamburg patient cohort in tumors (*n* = 199) and metastases (*n* = 65) with relative frequencies. (B) Representative images of analyzed metastasis tissue (upper images, left: ITIH5 expression = 0, right: ITIH5 expression = 1) and tumor tissue (lower images, left: ITIH5 expression = 2, right: ITIH5 expression = 3) from tissue microarray (TMA) samples. Scale bar 100 μm. (C) Significantly reduced ITIH5 expression in the Hamburg patient cohort in metastatic tissue (*n* = 65) compared to tumor tissue (*n* = 199) (Mann–Whitney *U* test; *P* < 0.05). (D) Kaplan–Meier plots showing cumulative survival of two subgroups, with PDAC patients expressing ITIH5 having longer progression‐free survival (PFS) than PDAC patients having lost ITIH5 expression (*n* = 189, events = 169, log‐rank test; *P* < 0.05). * < 0.05.

Next, ITIH5 protein expression was analyzed in pancreatic cancer patients using a well‐characterized C‐terminal polyclonal ITIH5 antibody that we had already validated by immunohistochemistry and Western blot previously [11] and re‐evaluated for this study (see Fig. S1A–E). Three independent tissue microarrays harboring pancreatic cancer tissues were stained with this antibody, including cohorts from the Institute of Pathology at the University Hospital Aachen (*n* = 41), from the University Hospital Hamburg (*n* = 508) and from the University Hospital Munich (*n* = 378). In the relatively small Aachen patient cohort, a tendency towards ITIH5 loss in the metastases (*n* = 19) was already detectable, but this was not yet significant. (Fig. S1G,J, *P* = 0.104). This observation was confirmed with clear significance in the Hamburg patient cohort, where the metastatic tissues showed complete loss of ITIH5 expression in 23.1% of cases and only weak ITIH5 expression in 61.5% of cases (Fig. 2A,B). There was significantly lower ITIH5 expression in metastases (*n* = 65, M = 1, SEM = 0.09) compared to tumor tissue (*n* = 199, M = 1.2, SEM = 0.06) (Fig. 2C, *P* < 0.05). Finally, assessing the Munich cohort including complete clinical follow‐up data demonstrated the impact of ITIH5 loss on patient survival. After dichotomizing the staining results into an ITIH5‐negative (complete loss of expression) and an ITIH5‐positive (weak to strong expression) group, a significantly longer cumulative survival time was shown in ITIH5‐expressing PDAC tissues (Fig. 2D, *P* < 0.05). Thus, immunohistochemistry showed significant loss of ITIH5 at the protein level during tumor progression towards metastasis, and loss of ITIH5 protein expression proved to be prognostically relevant as well.

### 3.4 Generation and characterization of stable ITIH5 gain‐of‐function models in human PDAC cell lines

**Fig. 3:**
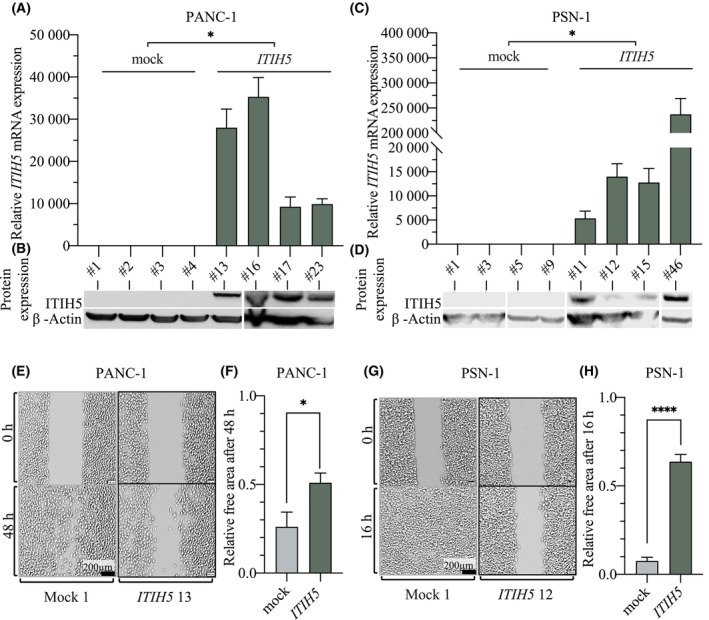
Forced ITIH5 re‐expression significantly impairs cell migration of PANC‐1 and PSN‐1 pancreatic cancer cell lines. (A–D) Gain‐of‐function models show significant re‐expression of ITIH5 in the ITIH5 clones on mRNA in triplicate measurements (A, C) and protein levels in single measurement (B, D) in both the PANC‐1 and PSN‐1 cell lines (A–D: *P* < 0.05, Mann–Whitney *U* test). Relative quantification of mRNA expression was based on the corresponding endogenous ITIH5 gene expression in the wild type cancer cell line. β‐Actin served as a loading control in the Western blot. Western blot overview was compiled from two blots. (E–H) Wound healing assay in pancreatic cancer cell lines. Representative images of the *in vitro* wound healing assay in mock and ITIH5 clones show reduced migratory capacity in ITIH5 re‐expressing cells in the two cell lines, in PANC‐1 after 48 h (E) and in PSN‐1 already after 16 h (G). Quantification of wound closure after a certain time shows a significant difference in migration in PANC‐1 (F) and PSN‐1 cell lines (H). Images are presented as they were processed and used for the measurement of the wound. PANC‐1 ITIH5 *P* < 0.05 and PSN‐1 ITIH5 *P* < 0.0001 compared to mock (Mann–Whitney *U* test). Scale bar 200 μm. Error bars are indicating the standard error of the mean (SEM), * < 0.05, **** < 0.0001.

Several pancreatic cancer cell lines were analyzed for their loss of endogenous ITIH5 expression aiming to select suitable cell lines to establish ITIH5 gain‐of‐function models. The PDAC cell lines PANC‐1, PSN‐1, MIAPaCa‐2, Dan‐G, BxPC‐3 and AsPC‐1 all showed no or only very low expression of endogenous ITIH5, both at the mRNA and protein levels (Fig. S1E). Based on previous studies of ITIH5 in PDAC and genetic features of the pancreatic cancer cell lines, PANC‐1 and PSN‐1 were chosen as suitable *in vitro* models [18, 55]. The gain‐of‐function models were generated for both cell lines by using the full‐length ITIH5 cDNA pBK‐CMV expression vector (referred to as ITIH5 clones) or an empty vector (referred to as mock clones) selecting stable clones, as described previously [16]. In both PANC‐1 and PSN‐1 lines, overexpression of ITIH5 was statistically significant at the mRNA and protein levels (Fig. 3A,C, *P* < 0.05, respectively and Fig. 3B,D, *P* < 0.05, respectively). We consequently successfully established ITIH5 re‐expressing cell models, which were used for subsequent experiments.

### 3.5 Forced ITIH5 re‐expression in human PDAC models impairs cell migration, but does not alter proliferation, apoptosis, or colony formation

Previous findings by the Welch group clearly suggested that *ITIH5* is a metastasis suppressor gene in PDAC [18]. To further investigate ITIH5 function, the PANC‐1 and PSN‐1 gain‐of‐function models grown from single clones were analyzed in functional *in vitro* assays. Measurement of migratory capacity by wound healing assay showed an inhibitory effect of forced ITIH5 re‐expression on migration capacity for both cell line models (Fig. 3E–H, Videos S1 and S2). Within a 48‐h observation period, PANC‐1 mock clones closed the wound significantly faster (74 ± 8% closed) than ITIH5‐overexpressing clones (49 ± 5% closed) (Fig. 3E,F, *P* < 0.05). In PSN‐1 clones, significantly faster migration was observed in the mock clones as early as 16 h, at which time the mock clones had already almost completely closed the wound (92 ± 2% closed), while at the same time, the ITIH5 clones had closed only 36 ± 4% of the wound (Fig. 3G,H, *P* < 0.001).

Furthermore, forced ITIH5 re‐expression had no effect on cell proliferation in the cell counting and XTT proliferation assays (Fig. S2A,B). The activity of the effector caspases 3 and 7 in the mock clones did not differ from that in the ITIH5 clones, either after 4 or after 16 h of induction, also in both lines. This indicates that ITIH5 has no effect on cell apoptosis (Fig. S2C). Additionally, no effect of ITIH5 re‐expression on the ability to form colonies was observed (Fig. S2D). Interestingly, the morphology of the clones seemed to be altered, especially in the formation of more compact colonies (Fig. S2E). Subsequently, we followed these two observations (changes in morphology and migration capacity) using more informative and sensitive methods.

### 3.6 ITIH5 regulates focal adhesion size and shape

It has been demonstrated in several studies that cell adhesion plays a crucial role in cell motility [45, 46, 50, 56, 57, 58, 59, 60]. Since the expression of ITIH5 in both PANC‐1 and PSN‐1 cells causes a clear impairment of their motility in the wound healing assay, we reasoned that ITIH5 may exert its action on cell motility via the regulation of cell adhesion. Cell adhesion is primarily controlled by the formation of focal adhesions (FAs); thus, it is reasonable to propose that ITIH5 may impact these subcellular structures.

**Fig. 4:**
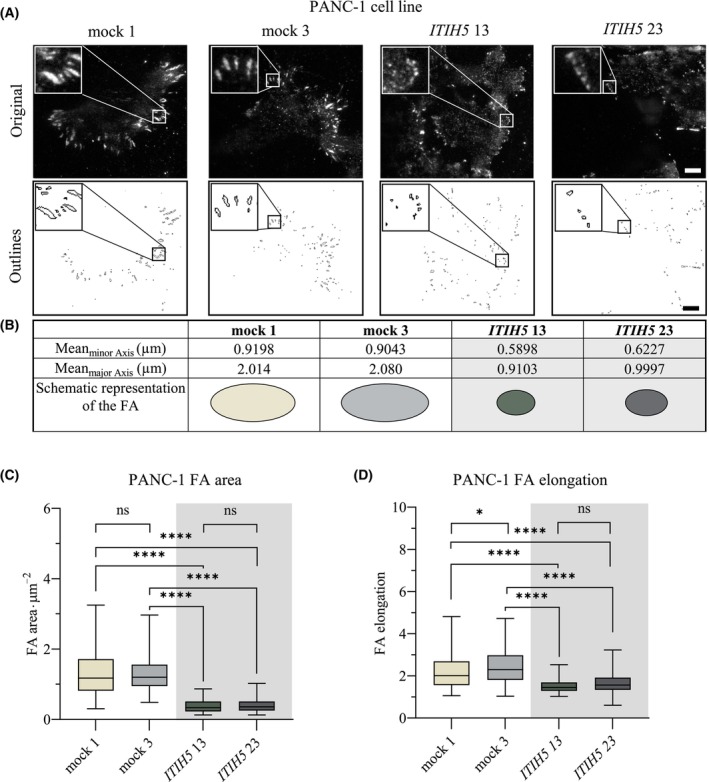
Re‐expression of ITIH5 reduces focal adhesion (FA) size and shape in PANC‐1 cell line. (A) Representative pictures of the focal adhesions in PANC‐1 ITIH5 and mock clones shown in the original images and FA outlines, including magnification boxes for each. Scale bar 10 microns. (B) Mean minor and mean major axis of the focal adhesions in analyzed clones with schematic representation. (C) FA area is significantly reduced in PANC‐1 ITIH5 clones compared to mock clones [One‐Way Analysis of Variance (ANOVA), Turkey's multiple comparison test]. (D) FAs are less elongated in PANC‐1 ITIH5 compared to mock clones (ANOVA, Turkey's multiple comparison test; *n*_ITIH5 13_ = 135, *n*_ITIH5 23_ = 127, *n*_mock 1_ = 131, *n*_mock 4_ = 145). Error bars are indicating the standard error of the mean (SEM), ns = not significant, * < 0.05, **** < 0.0001.

**Fig. 5:**
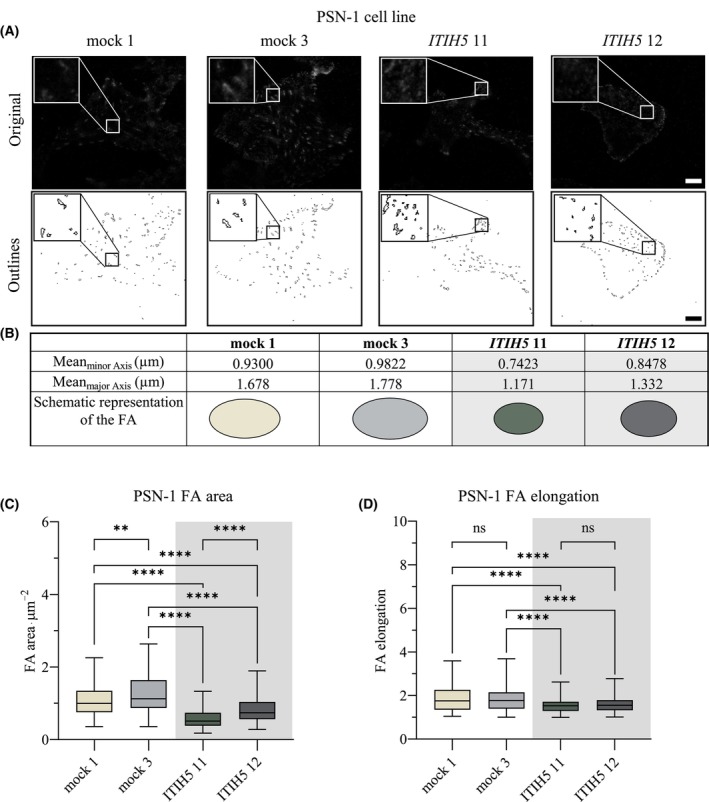
Re‐expression of ITIH5 reduces focal adhesion (FA) size and shape in PSN‐1 cell line. (A) Representative pictures of the focal adhesions in PSN‐1 ITIH5 and mock clones shown in the original images and FA outlines, including magnification boxes for each. Scale bar 10 microns. (B) Mean minor and mean major axis of the focal adhesions in analyzed clones with schematic representation. (C) FA area is significantly reduced in PSN‐1 *ITIH5* clones compared to mock clones [One‐Way Analysis of Variance (ANOVA), Turkey's multiple comparison test]. (D) FAs are less elongated in PSN‐1 *ITIH5* compared to mock clones (ANOVA, Turkey's multiple comparison test; *n*_ITIH5 11_ = 146, *n*_ITIH5 12_ = 157, *n*_mock 1_ = 121, *n*_mock 3_ = 175). Error bars are indicating the standard error of the mean (SEM), ns = not significant, ** < 0.01, **** < 0.0001.

**Fig. 6:**
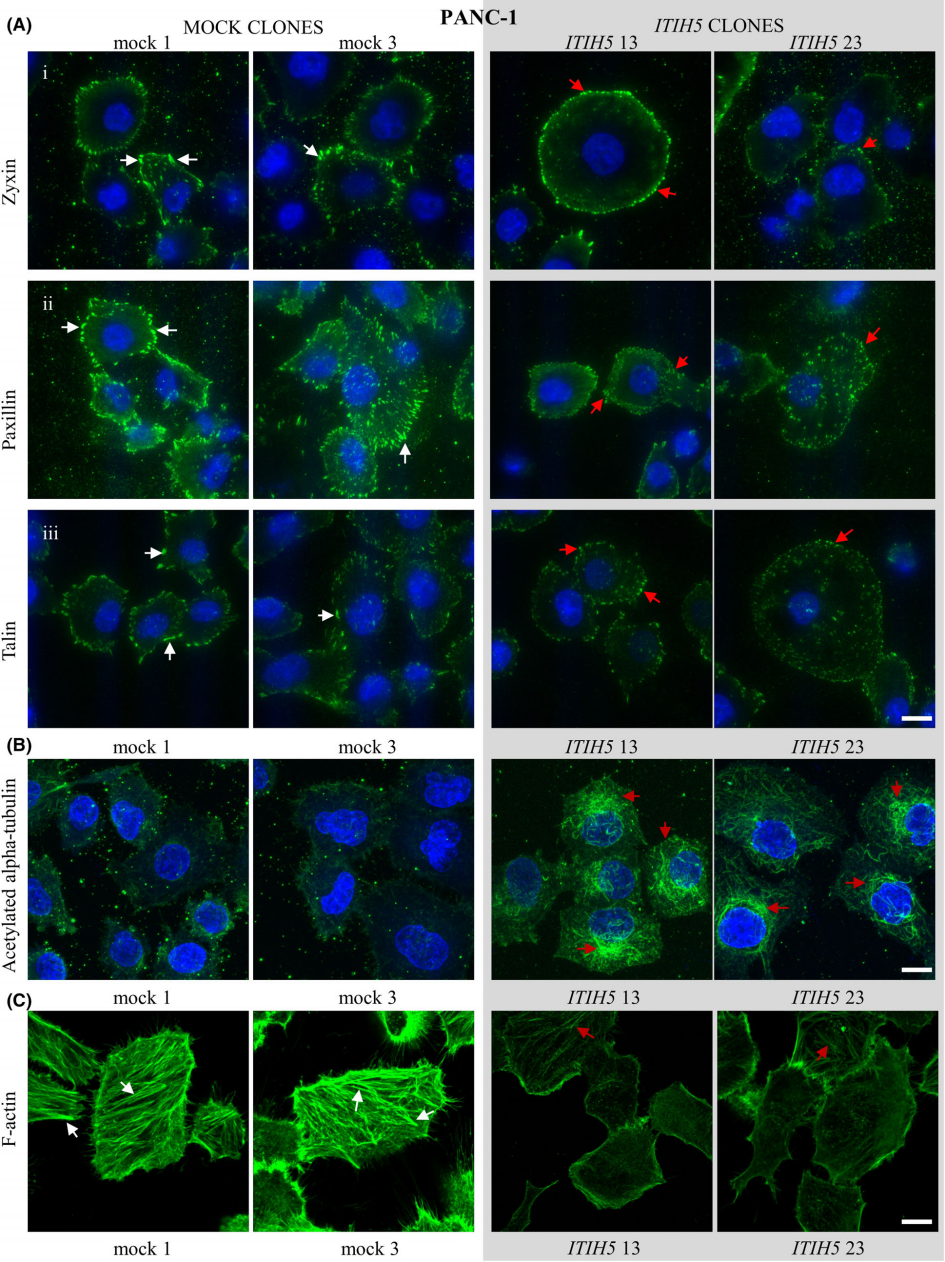
Focal adhesion (FA) protein distribution and more stable microtubules accompanied by decreased F‐Actin in ITIH5‐expressing PANC‐1 clones. (A) PANC‐1 mock clones and ITIH5‐expressing clones were fixed and then stained with antibodies against zyxin (row i), paxillin (row ii) and talin (row iii) to visualize focal adhesions. Mock clones (white arrows) show larger and more elongated focal adhesions than focal adhesions in ITIH5‐expressing clones (red arrows). (B) PANC‐1 clones were fixed and then stained with antibodies against acetylated alpha‐tubulin. Note the higher levels of acetylated alpha‐tubulin in ITIH5 clones (red arrows) that label a large portion of the microtubule cytoskeleton. By contrast, very little acetylated alpha‐tubulin can be observed in control PANC‐1 clones. DAPI was used to visualize nuclei. (C) PANC‐1 clones were fixed and then stained with antibodies against F‐Actin. Note the better‐developed F‐Actin cytoskeleton characterized by more numerous and thicker stress fibers in mock clones (white arrows) compared to ITIH5 clones (red arrows). Representative images from three independent experiments for each clone are shown for all stainings. Scale bar: 10 μm.

**Fig. 7:**
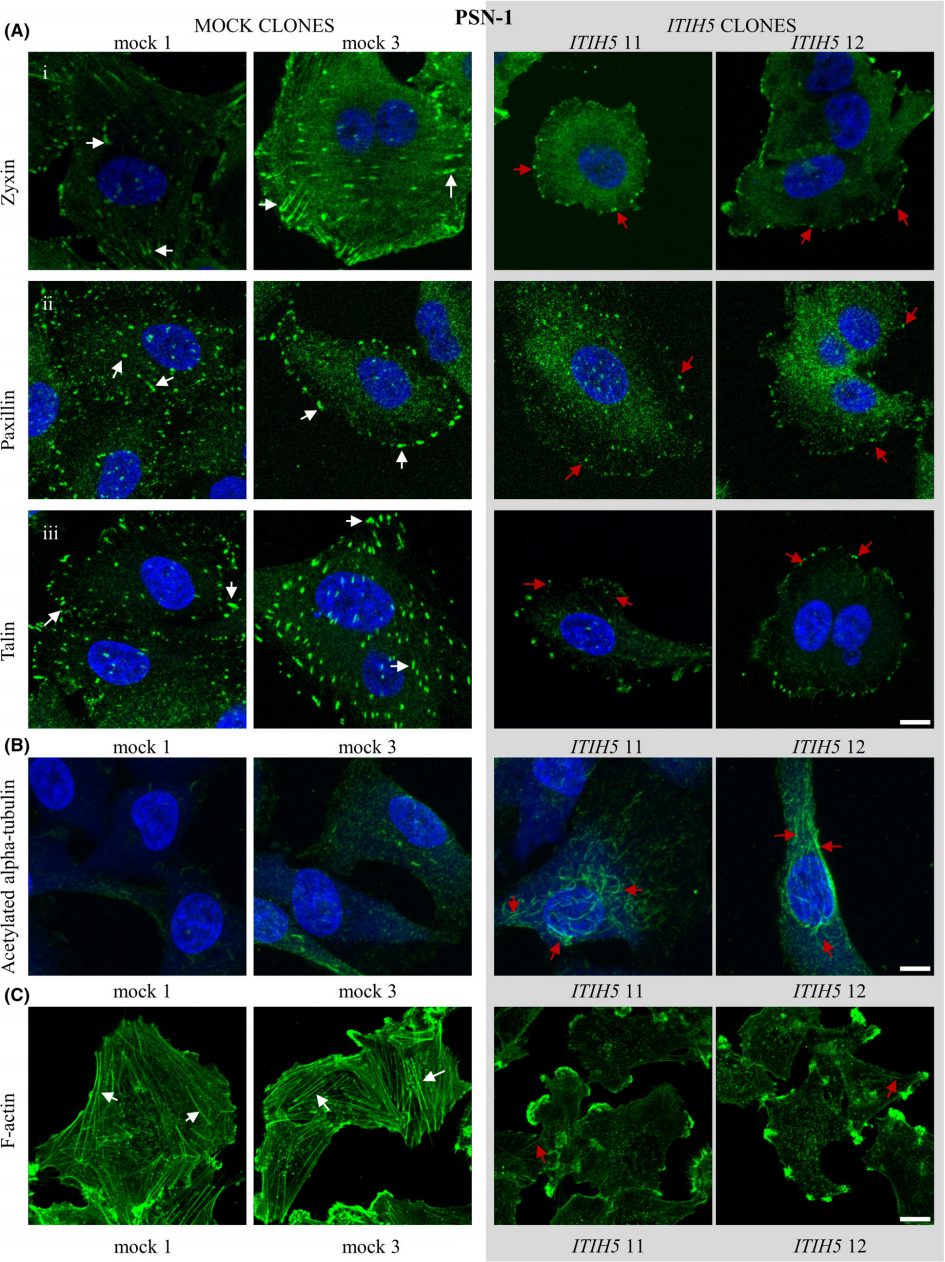
Focal adhesion (FA) protein distribution and more stable microtubules accompanied by decreased F‐Actin in ITIH5‐expressing PSN‐1 clones. (A) PSN‐1 mock clones and ITIH5‐expressing clones were fixed and then stained with antibodies against zyxin (row i), paxillin (row ii) or talin (row iii) to visualize focal adhesions. Mock clones (white arrows) show larger and more elongated focal adhesions than focal adhesions in ITIH5‐expressing clones (red arrows). (B) PSN‐1 clones were fixed and then stained with antibodies against acetylated alpha‐tubulin. Note the higher levels of acetylated alpha‐tubulin in ITIH5 clones (red arrows) that label a large portion of the microtubule cytoskeleton. By contrast, very little acetylated alpha‐tubulin can be observed in control PSN‐1 clones. DAPI was used to visualize nuclei. (C) PSN‐1 clones were fixed and then stained with antibodies against F‐Actin. Note the better‐developed F‐Actin cytoskeleton characterized by more numerous and thicker stress fibers in mock clones (white arrows) compared to ITIH5 clones (red arrows). Representative images from three independent experiments for each clone are shown for all stainings. Scale bar: 10 μm.

To test our hypothesis, we labeled PANC‐1 and PSN‐1 ITIH5‐expressing and mock clones with an antibody against vinculin to visualize FAs and imaged these structures using TIRF microscopy (Figs 4A and 5A). Afterwards, we analyzed static FA properties such as area, major and minor axis and elongation using dedicated segmentation algorithms [48, 49]. In both PANC‐1 and PSN‐1 cells, ITIH5‐expressing clones exhibited smaller and more rounded FAs compared to control clones (Figs 4B and 5B). Forced re‐expression of ITIH5 caused a reduction of FA area from 1.307 ± 0.034 μm^2^ to 0.396 ± 0.013 μm^2^ in PANC‐1 cells and from 1.185 ± 0.02817 μm^2^ to 0.7055 ± 0.01905 μm^2^ in PSN‐1 cells as well as a reduced elongation of FAs (Figs 4C,D and 5C,D). Likewise, the major and minor axes were also reduced in cells expressing ITIH5 (Figs 4B and 5B). To gain a more comprehensive view on the FAs, we visualized further key components of FAs with antibodies against zyxin, paxillin, and talin (Figs 6A and 7A). Applying these more detailed stainings, similar morphological changes in terms of smaller and more rounded FAs could be noted, pointed out in Figs 6A and 7A with white arrows in mock and red arrows in ITIH5 clones. Furthermore, a staining with antibodies against acetylated alpha‐tubulin showed considerably higher levels of acetylated alpha‐tubulin in ITIH5 clones compared to mock clones (Figs 6B and 7B, Fig. S3A,B). Finally, a staining using antibodies against F‐actin displayed a less‐developed F‐actin cytoskeleton characterized by fewer and thinner stress fibers in ITIH5 clones compared to mock clones in both cell lines (Figs 6C and 7C, Fig. S3C,D). Our observation of reduced FA size in ITIH5 re‐expressing models clearly suggests that ITIH5 regulates cell migration via the formation of FA and, moreover, influences cytoskeletal structure.

### 3.7 Forced ITIH5 re‐expression in human PDAC models reduces intracellular kinase activity related to focal adhesion

**Fig. 8:**
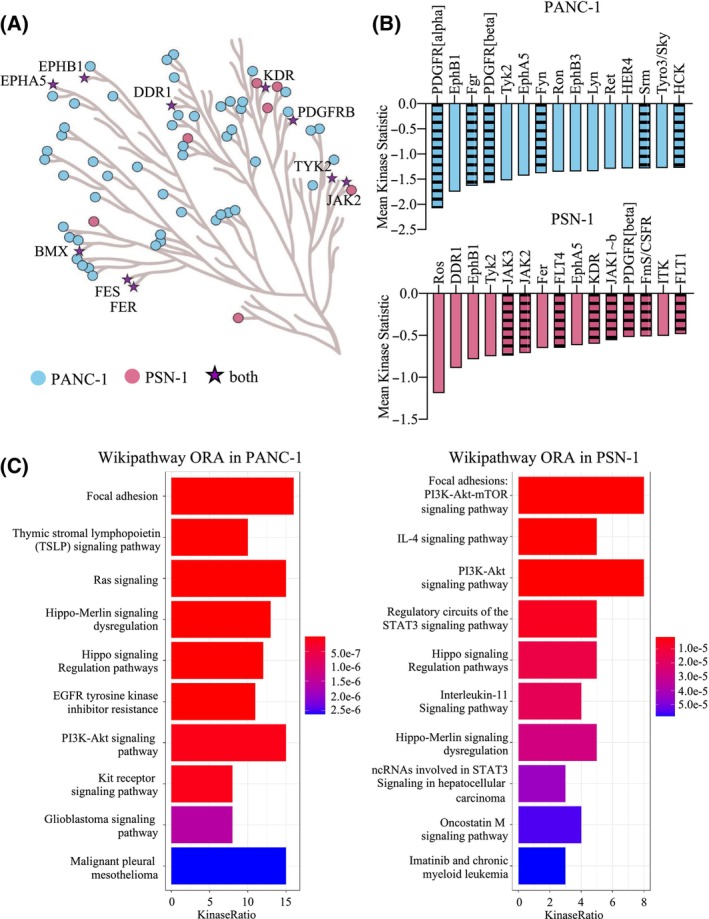
ITIH5 re‐expression in pancreatic cancer cells reduces activity of Focal adhesion (FA) kinases and important signaling pathways. (A) Comparison of downregulated tyrosine kinases in PANC‐1 and PSN‐1 ITIH5 re‐expressing cells, visualized in a coral tree diagram. Peptide tyrosine kinases (PTKs) downregulated in both cell lines are illustrated by stars. (B) Bar charts showing the top 15 downregulated PTKs in PANC‐1 and PSN‐1 ITIH5 re‐expressing cells. Striped columns mark PTKs related to focal adhesion. (C) Bar charts visualizing the significance levels of the top 10 WikiPathways pathways based on the PTKs with significant differences in PANC‐1 and PSN‐1 cells re‐expressing *ITIH5* compared to mock using over‐representation analysis (ORA). Significant levels (adjusted *P*‐values) are color‐coded from blue to red.

In order to understand the biochemical effects that are induced by forced ITIH5 re‐expression, the PANC‐1 and PSN‐1 gain‐of‐function models were subjected to PTK activity profiling (see Tables S7 and S8). It could be observed that the activity of many PTKs was strongly reduced in both PDAC cell lines re‐expressing ITIH5, as visualized in a coral tree diagram (Fig. 8A). In the PANC‐1 cell line ITIH5 re‐expression showed a more comprehensive suppressive effect on tyrosine kinases but there was considerable overlap in the affected PTKs between the two pancreatic cancer cell lines (Fig. 8A,B). Strikingly, ORA of WikiPathways for the kinases with significant differences revealed that in both cell lines the most downregulated kinases are related to focal adhesion in a direct manner (Fig. 8B,C), or indirectly via Ras, Hippo or in particular the PI3K/Akt signaling pathway [61, 62] (Fig. 8C). Coherently, we observed a reduction in the activity of PTKs, particularly those associated with FAs, after forced re‐expression of ITIH5. This data provides further evidence that ITIH5's metastasis suppressive action in PDAC may be mediated by alterations in cytoskeletal structures such as FAs and microtubules.

## 4 Discussion

Pancreatic cancer remains a challenging disease with poor prognosis, demanding a better understanding of this malignancy to identify more effective strategies for diagnosis and treatment. At present, only patients with regionally limited PDAC, and the possibility to undergo margin‐negative resection, have an opportunity for curative therapy [26, 31]. However, even with this procedure, long‐term survival of patients that underwent surgery remains below 20% [63]. Therefore, chemotherapy with either FOLFIRINOX or gemcitabine with nab‐paclitaxel is recommended for all stages of PDAC, whether localized, locally advanced, or metastatic cases [30, 31]. Unfortunately, in approximately 70% of cases of metastatic pancreatic cancer, this standard first‐line chemotherapy does not result in a tumor response [64]. Resistance to chemotherapeutic agents is primarily attributed to the strong desmoplastic reaction classically occurring in PDAC [65]. Desmoplastic stroma acts as a barrier, impeding proper vascularization and consequently limiting e.g., drugs' exposure and effectiveness [66]. In addition, the heterogeneity of PDAC might contribute to the inadequate therapy response [25]. Approaches to address the chemotherapeutical resistance by e.g. subgrouping PDAC and developing promising personalized therapies are currently in development. For instance, there are strategies targeting inherited and somatic mutations in driver genes such as *BRCA1* and *BRCA2* mutations, often still in the context of clinical trials [30, 31, 67]. However, since these genetic alterations only occur in single digit percentages of PDAC, it remains important to continue the research on PDAC relevant genes, including tumor suppressor genes. Understanding their underlying mode of action may allow scientists to derive new concepts to treat this devasting cancer. In this regard, *ITIH5* may constitute a promising candidate as it is known to be a potent tumor suppressor gene in several tumor entities [8, 11, 13], while in pancreatic cancer *ITIH5* has been functional validated in depth as a metastasis suppressor gene [18, 19]. Those prior work indicates that expression of ITIH5 gets lost during cancer progression leading to an unfavorable prognosis [8, 11, 13] particularly in less differentiated tissue for example in CIMP‐positive colon [13], basal‐like breast [16] and basal/squamous‐like bladder cancer [33]. *ITIH5* promoter hypermethylation is the key mechanism for its expression loss in various tumor entities [8, 11, 13, 14, 21]. This makes *ITIH5* a convincing class 2 tumor suppressor gene (C2TSG) or DNA methylation driver gene (DNAme driver) in these tumor entities [22, 23]. C2TSGs may have potential in future personalized therapies in treating particularly challenging malignancies, where typical druggable driver mutations are not present [24]. This recently proposed concept is based on small molecule mimetics and involves the identification and validating of drug candidates that phenotypically mimic proteins encoded by epigenetically silenced tumor suppressor genes [24]. In PDAC, C2TSGs could become of great interest as promoter DNA methylation, among other epigenetic changes, is discussed to be the driving force that alters gene expression and significantly influences malignant PDAC phenotypes [25].

In the present study, we show for the first time that *ITIH5* is subject to significant promoter hypermethylation in pancreatic cancer based on TCGA dataset as well as on our own FFPE tissue cohort analysis. Like in other solid tumor entities analyzed by our group, the TCGA cohort showed a highly significant hypermethylation of the *ITIH5* promoter region in pancreatic cancer. In particular, the region −777 to −1002 bases upstream of the transcription start site exhibited highly significant DNA hypermethylation. This corresponds very well to our previous work on the characterization of the *ITIH5* gene promoter in bladder cancer, where significant hypermethylation was associated with shorter overall survival [41]. Our results of the TCGA cohort were verified by promoter DNA pyrosequencing of our own FFPE patient population. Here, we found significant *ITIH5* promoter hypermethylation in tumor and metastatic tissue compared to normal pancreatic acinar tissue. Analysis of the mRNA from the FFPE tissue cohort showed a concomitant inverse correlation between hypermethylation and *ITIH5* expression, supporting the assumption of a transcriptional regulatory effect on ITIH5 in PDAC. Loss of ITIH5 expression on protein level has been demonstrated by IHC in various entities recently [8, 11, 12, 13, 14, 21, 68], including pancreatic cancer [18, 19]. In this context, Young et al. stated that loss of ITIH5 in pancreatic cancer may be an early event occurring in the primary tumor, as no significant additional reduction of ITIH5 protein expression was observed in metastatic tissue compared to primary tumors [19]. Concordantly, we saw in our PDAC patient cohort a significant promotor methylation taking place equally in tumor and metastasis compared to normal tissue [69]. However, using our extensively validated C‐terminal ITIH5 antibody, we observed the major loss of ITIH5 protein in the step from the primary tumors to the metastases. This variation in the time point of ITIH5 protein loss between the two IHC studies might be due to the well‐known heterogeneity of pancreatic cancer [70]. However, both studies demonstrate loss of ITIH5 in malignant pancreatic tissue and a significant survival benefit of the ITIH5 expressing cohort [18].

The influence of ITIH5 overexpression on tumor cell characteristics has been described in many different entities. While ITIH5 shows several tumor suppressive properties, e.g. on proliferation, apoptosis, and migration in other malignancies like breast cancer [8, 9, 10, 11, 12, 13, 14], these properties are restricted to migration inhibition in pancreatic cancer cells, consistent with previous results [18, 19]. Hence, our observations further support the idea first formulated by the Welch group that *ITIH5* is a typical metastasis suppressor gene in pancreatic cancer. The observed alterations in FA of pancreatic cancer cells after forced ITIH5 expression suggest a potential mechanism causing suppression of migration and thus metastasis capability. It has been long established that cell migration requires the coordination, in time and space, of cytoskeleton‐dependent processes such as the formation of FAs at the cell's front and the disassembly at its rear [71, 72]. The robust inhibitory effect of ITIH5 on cell migration suggests that one or more cytoskeleton‐dependent processes required for cell migration may be altered. The analysis of FAs shows that forced ITIH5 re‐expression results in smaller and rounded FAs, similar to our previous findings on ITIH5 re‐expression in bladder cancer [33]. The speed of cell movement exhibits a biphasic relationship to the size of FAs, meaning that fast moving cells exhibit larger and more elongated FAs [73]. It is therefore conceivable that the metastasis‐suppressing effect of ITIH5 occurs via the impairment of cell adhesion. In this context, it is interesting to note that high mechanical stress promotes stress fiber formation and cell motility in pancreatic cancer cells (including the PANC‐1 cells used in our study) via the activation of PI3K/Akt, MAPK, and JNK signaling pathways [74]. Similarly, in breast cancer cells mechanical stress increases cellular tension inducing the formation of stress fibers, and focal adhesions and promoting cell motility [75]. Thus, our data are consistent with these studies in that the re‐expression of ITIH5 causes a robust reduction of stress fibers and focal adhesion formation (i.e., very low mechanical cellular stress) as well as impairment of signaling pathways such as PI3K/Akt thus resulting in impairment of cell motility.

Although the precise understanding of the molecular mechanisms underlying the impact of ITIH5 on focal adhesion function and cell migration is beyond the scope of this study, it is remarkable that many kinases involved in FA function are robustly hypo‐phosphorylated upon re‐expression of ITIH5. Along with this line of reasoning, we have shown a clear increase of microtubule acetylation (i.e., more stable microtubules) in cells re‐expressing ITIH5. Since highly dynamic microtubules are required for FA turnover [76, 77], we speculate that an additional mechanism of action of ITIH5 could involve the impairment of the functional axis between microtubules and FAs.

Finally, our tyrosine kinase activity profile revealed differentially activated signaling pathways upon re‐expression of ITIH5, providing potential insights into the mechanisms behind ITIH5's impact on migration, FAs, and cytoskeletal elements. Besides its suppressive effect on kinases directly associated with FAs molecules, our PTK analysis brings attention to the potential influence of ITIH5 on other molecular pathways, like for example PI3K‐AKT signaling. This pathway plays a key role in regulating cell migration and is known to be dysregulated in many cancer types [78, 79, 80]. Especially in pancreatic cancer, a signal‐independent activation of the PI3K/AKT pathway is common by activating mutations in KRAS [81] and PI3K subunits [82, 83] or the upregulation of receptor tyrosine kinases (RTKs) [84]. PI3K/AKT is also known to interact with cellular components and cytoskeletal proteins like actin filaments and microtubules [85] thus affecting actin dynamics [86] and cytoskeletal structure [86, 87]. Future investigations will certainly be centered on precisely characterizing the signaling pathways regulated by ITIH5.

How the intriguing, yet still incomplete insights into the mechanisms of ITIH5 action in pancreatic cancer can be applied in a clinical context is for future determination. ITIH5 could emerge as a compelling tumor suppressor protein in pancreatic cancer, whose functional restoration through specific DNA demethylation [25], gene therapy [88] or small molecule mimetic [24] could become an important therapeutic target in future personalized medicine.

## 5 Conclusions

The results of our study shed light on the potentially crucial role of ITIH5 in pancreatic cancer progression and metastasis. The observed robust hypermethylation of the ITIH5 promoter in pancreatic tumors and metastases highlights its potential importance as a prognostic marker for unfavorable overall survival in patients with PDAC. Characterization of stable ITIH5 gain‐of‐function models in human PDAC cell lines provided valuable insights into the functional consequences of ITIH5 re‐expression. These include changes in the size and shape of focal adhesion as well as a reduction in cell migration capacity, which may explain the impaired metastatic potential of such cells as shown in a previous study. Furthermore, the discovery that ITIH5 interferes with several oncogenic PTK signaling pathways highlights its potential as a therapeutic tumor suppressor protein target to mitigate pancreatic cancer, e.g. by mimetic drugs that can compensate for ITIH5 loss.

## Conflict of interest

The authors declare no conflict of interest.

## Author contributions

Conceptualization: JK, AS, ED. Data collection: JK, JH, LH, JB, NO‐B, TK, RS, GS, SAH, MH, MR, FS, SLM, EPCV, AA‐H, DJ. Formal analysis: JK, SV, AS. Resources: LH, SLM, NO‐B, TK, RS, GS, JB, DJ. Visualization: JK, JH, AS, MR, SLM, EPCV. Writing – original draft: JK. Writing – review and editing, JK, AS, DJ, ED. Supervision: ED. Funding acquisition: ED. All authors read and approved the final manuscript.

### Peer review

The peer review history for this article is available at https://www.webofscience.com/api/gateway/wos/peer‐review/10.1002/1878‐0261.13609.

## Supporting information


**Fig. S1.** ITIH5 antibody validation.
**Fig. S2.** ITIH5‐overexpression does not alter growth, apoptosis and colony formation of PANC‐1 and PSN‐1 cells.
**Fig. S3.** More stable microtubule and reduced F‐actin formation in ITIH5 overexpressing cells.

**Table S1.** Sample Id's of the TCGA pancreatic Adenocarcinoma data set.
**Table S2.** Description of the patient cohort from Aachen with ductal adenocarcinomas of the pancreas.
**Table S3.** Description of the patient cohort from Hamburg with ductal adenocarcinomas of the pancreas.
**Table S4.** RT‐PCR primer sequences and PCR conditions.
**Table S5.** Pyrosequencing Primer sequences and PCR conditions.
**Table S6.** Primary and secondary antibodies for Western Blot.
**Table S7.** Results of kinase analysis carried out within the kinase assay in PANC‐1.
**Table S8.** Results of kinase analysis carried out within the kinase assay in PSN‐1.

**Video S1.** Live imaging of PANC‐1 cells in the wound healing assay.

**Video S2.** Live imaging of PSN‐1 cells in the wound healing assay.

## Data Availability

The data supporting the study findings are available from the authors upon reasonable request.
