# Supplementary material for: ITIH5 as a multifaceted player in pancreatic cancer suppression, impairing tyrosine kinase signaling, cell adhesion and migration
Source: Mol Oncol. 2024 Feb 20;18(6):1486–509. doi: 10.1002/1878-0261.13609 (PMC11161730; doi:10.1002/1878-0261.13609)
Supplement: Supplementary file 1 — Fig. S1. ITIH5 antibody validation. Fig. S2. ITIH5‐overexpression does not alter growth, apoptosis and colony formation of PANC‐1 and PSN‐1 cells. Fig. S3. More stable microtubule and reduced F‐actin formation in ITIH5 overexpressing cells. [file MOL2-18-1486-s003.pdf]

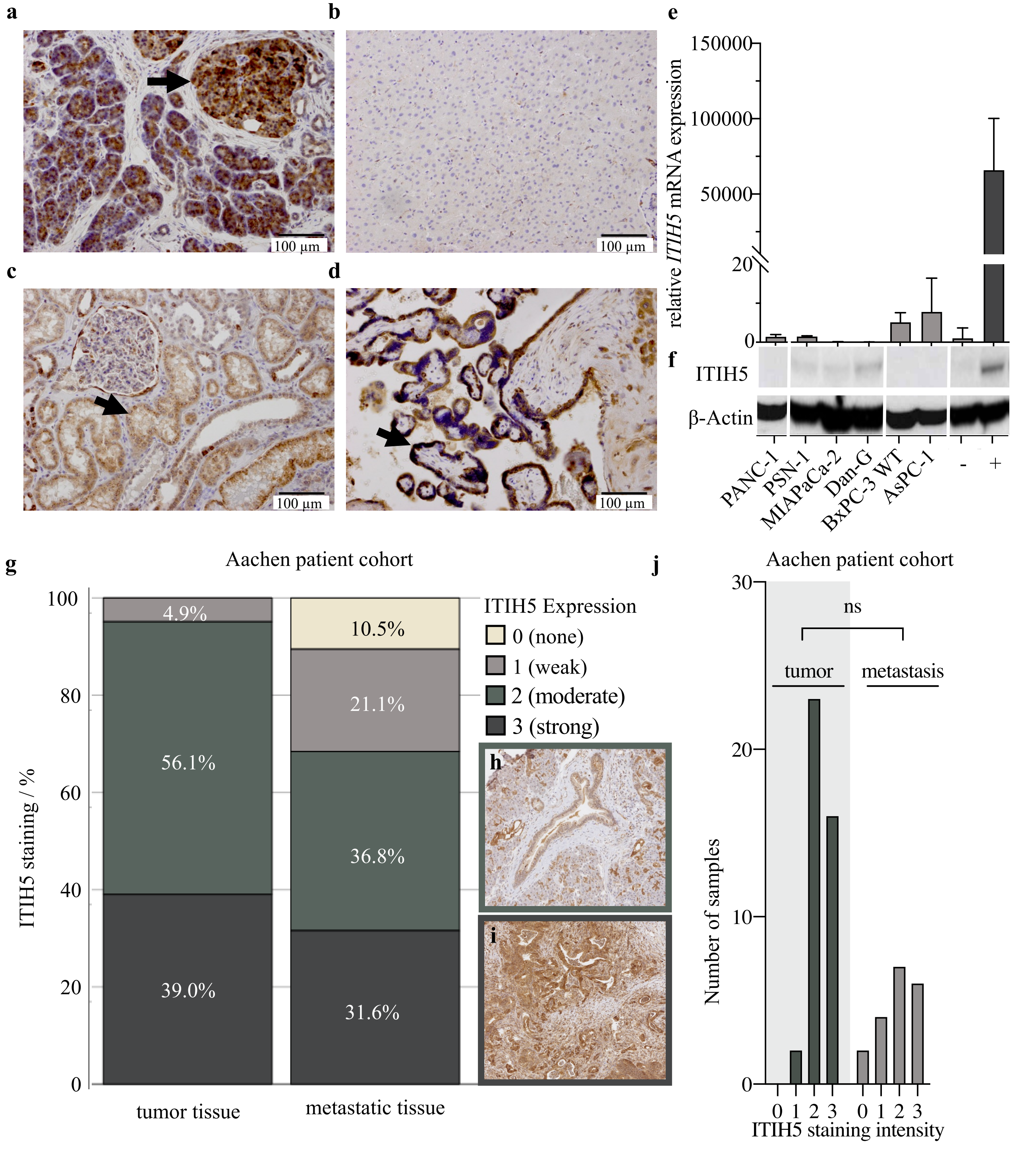

Supplementary Figure S1: **ITIH5 antibody validation. Staining of Aachen cohort with no significant difference in ITIH5 protein expression. a-d**: Representative immunohistochemistry images of the human ITIH5 antibody (Pineda 3, polyclonal rabbit antibody; 1:200 dilution) shows the characteristic ITIH5 expression patterns. ITIH5 expression pattern in human pancreatic tissue (**a**) with strong ITIH5 expression in islets of Langerhans cells (arrow). Human liver tissue does not express ITIH5 serving as negative control (**b**). ITIH5 expression pattern in human kidney tissue (**c**) with moderately stained tubular epithelium (arrow). ITIH5 expression pattern in human placenta tissue served as positive control (**d**) with strongly stained trophoblasts cells (arrow). **e,f**: ITIH5 expression in different human PDAC cell lines based on mRNA (**e**) and protein levels (**f**). Relative quantification of mRNA expression was based on the corresponding endogenous ITIH5 gene expression in the wild type. β-Actin served as a loading control in the Western blot. Western blot overview was cut together. **g**: Distribution of ITIH5 expression in the Aachen patient cohort within tumor tissue samples ($n = 41$) and metastatic tissue samples ($n = 19$) with relative frequencies. **h, i**: Representative images of analyzed tumor and metastatic tissue from TMA samples. ITIH5 expression = 2 (**h**), ITIH5 expression = 3 (**i**). **j**: No significant difference in ITIH5 expression in the Aachen patient cohort in metastatic tissue ($n = 19$) compared with tumor tissue ($n = 41$) (Mann-Whitney U test; $p = 0.104$). (ns = not significant)

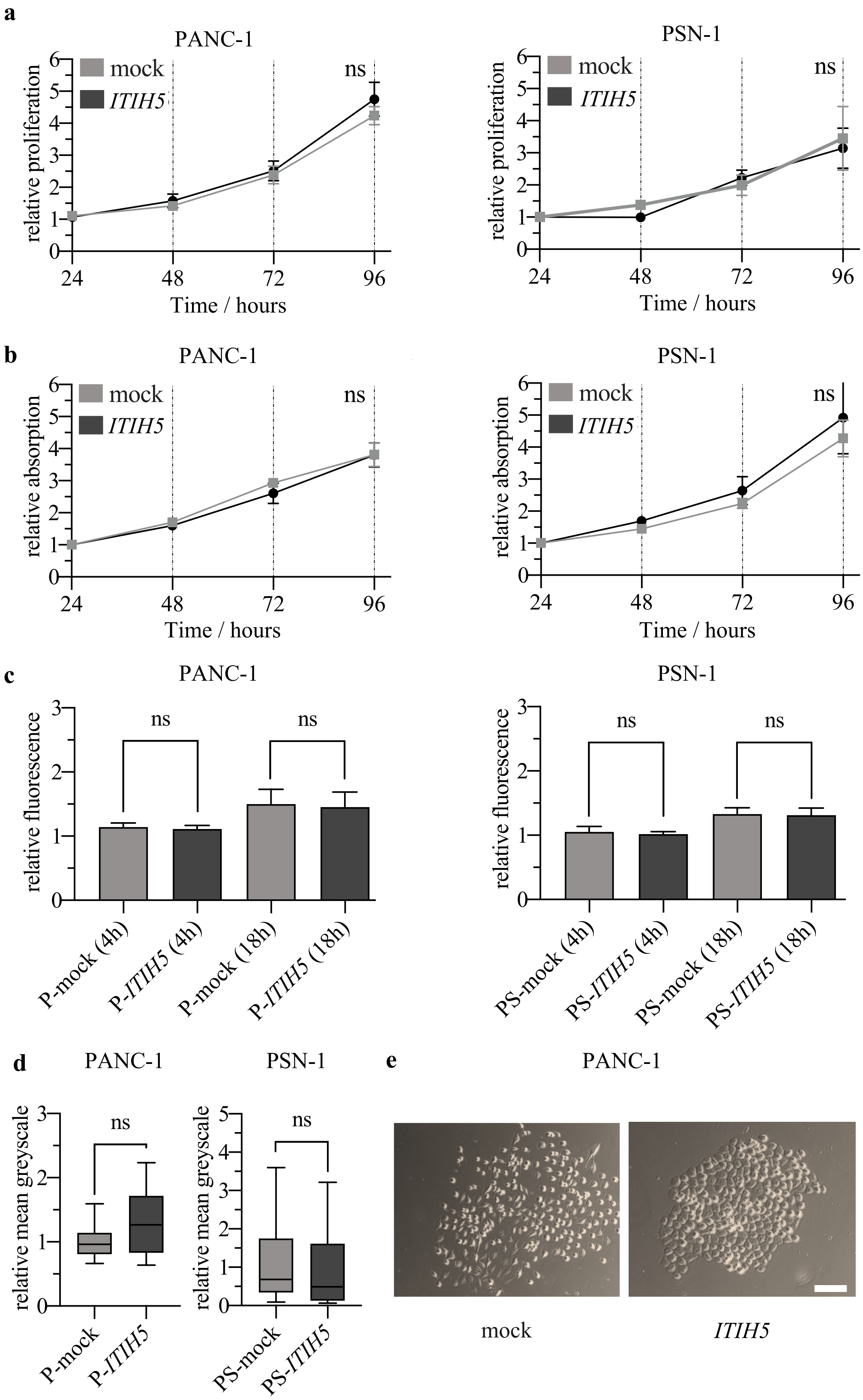

Supplementary Figure S2: *ITIH5*-overexpression does not alter growth, apoptosis and colony formation of PANC-1 and PSN-1 cells. The growth curves of the cell counting assay of the PANC-1 (**a**) and PSN-1 cell line (**b**) show no significant difference in proliferation between *ITIH5* and mock clones. The XTT assay shows no significant difference in cell metabolism between *ITIH5* and mock clones (**c,d**). The induction of apoptosis shows no significant difference between *ITIH5* and mock clones in the PANC-1 (P, **i**) and in the PSN-1 cell line (PS, **j**), neither after 4 h of incubation nor after 18 h. No significant differences were observed between *ITIH5* and mock clones in the colony formation assay in the PANC-1 (**k**) and the PSN-1 cell line (**m**). The Mann-Whitney U test was used for the comparisons. (P = PANC-1, PS = PSN-1, ns = not significant).

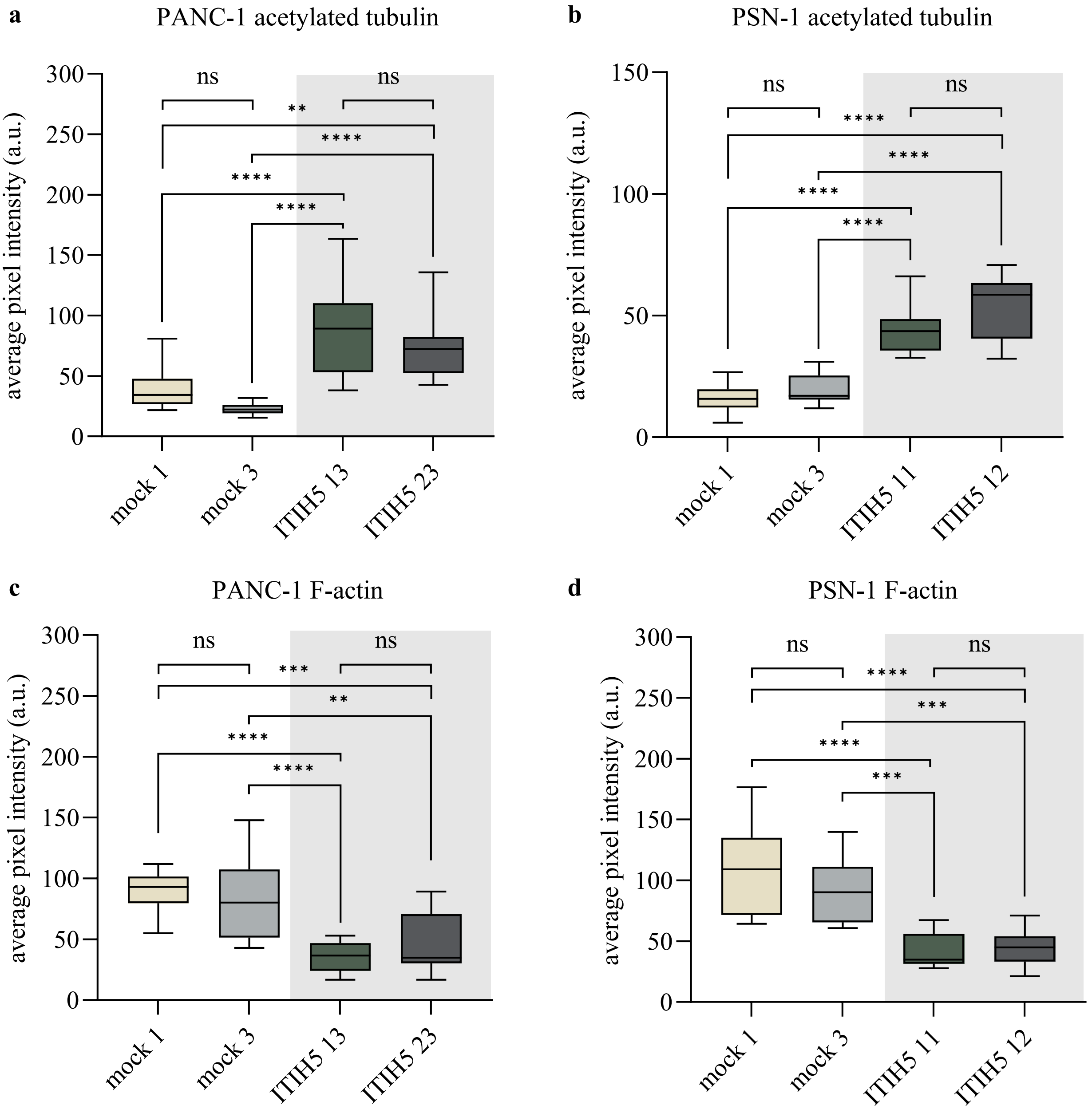

Supplementary Figure S3: More stable microtubule and reduced F-actin formation in *ITIH5* overexpressing cells. a,b: Levels of acetylated alpha-tubulin are significantly increased in ITIH5 overexpressing clones in PANC-1 (a) as well as in PSN-1 cell line (b) compared to mock clones. c,d: F-actin formation is significantly reduced in ITIH5 overexpressing PANC-1 (c) and PSN-1 clones (d) compared to mock clones. ns = not significant, * < 0.05, ** < 0.01, *** < 0.001 **** < 0.0001.
