# Supplementary material for: ITIH5 as a multifaceted player in pancreatic cancer suppression, impairing tyrosine kinase signaling, cell adhesion and migration
Source: Mol Oncol. 2024 Feb 20;18(6):1486–509. doi: 10.1002/1878-0261.13609 (PMC11161730; doi:10.1002/1878-0261.13609)
Supplement: Supplementary file 3 — Video S1. Live imaging of PANC‐1 cells in the wound healing assay. [file MOL2-18-1486-s004.zip › Additional file 3_Supplementary Video 1_Caption.docx]

**Supplementary video 1: Live imaging of PANC-1 cells in the wound healing assay.** Control PANC-1 clones (mock 1 and mock 3, upper side) and PANC-1 cells stably expressing ITIH5 (ITHI5 13 and ITH5 23, lower side) were filmed at 37°C, 5% CO_2_ for 20 hours. Images were acquired every 5 minutes. Note the significantly slower motility of the PANC-1 cells expressing ITIH5 failing to close the scratch. The playback speed is approximately 6.000 times the original speed.
